# Paroxysmal nocturnal hemoglobinuria with a positive Coombs test presenting as acute kidney injury: a case report

**DOI:** 10.3389/fmed.2026.1778834

**Published:** 2026-05-08

**Authors:** Luyao Jiang, Tianxi Chen, Linlin Xu, Miaojun Shi, Wen Huang

**Affiliations:** 1 Department of Rehabilitation Medicine, The First People’s Hospital of Yongkang, Jinhua, Zhejiang, China; 2 Department of Nephrology, The First People’s Hospital of Yongkang, Jinhua, Zhejiang, China; 3 Department of Nephrology, The Second Affiliated Hospital and Yuying Children’s Hospital of Wenzhou Medical University, Wenzhou, Zhejiang, China

**Keywords:** acute kidney injury, case report, Coombs test, eculizumab, paroxysmal nocturnal hemoglobinuria

## Abstract

This report presents the case of a 60-year-old woman who was admitted with symptoms of fatigue and poor appetite. Laboratory investigations revealed severe acute kidney injury (AKI), indicated by a serum creatinine level of 2,065 μmol/L, necessitating the initiation of emergency hemodialysis. The patient also exhibited hemolytic anemia (hemoglobin, 69 g/L), thrombocytopenia (platelet nadir, 45 × 10^9^/L), venous thrombosis, a positive direct antiglobulin test (DAT), and a low absolute reticulocyte count. Conventional flow cytometry (red blood cells and neutrophils CD55/CD59) did not detect a paroxysmal nocturnal hemoglobinuria (PNH) clone. Renal biopsy revealed hemoglobin cast nephropathy and intrarenal venous thrombosis. Subsequent bone marrow evaluation, high-sensitivity flow cytometry (FLAER method), and supportive PIGA sequencing findings established the diagnosis of PNH. Treatment with eculizumab led to hematologic remission and progressive renal recovery. This case underscores the importance of considering PNH in patients with severe AKI and hemolysis, even when initial conventional flow cytometry is negative.

## 1 Introduction

Paroxysmal nocturnal hemoglobinuria (PNH) is an acquired clonal hematopoietic stem cell disorder caused by somatic *PIGA* mutations, characterized by complement-mediated hemolysis, thrombosis, and bone marrow failure (1). While mild or chronic renal impairment is a known complication of PNH (2), severe acute kidney injury (AKI) presenting as the initial and predominant clinical feature is exceedingly rare. Such a life-threatening renal presentation often obscures the underlying hematologic etiology. The diagnostic challenge intensifies when atypical features, such as a positive DAT, are present or when confounding factors lead to initially negative or non-diagnostic results on standard flow cytometry.

We report a rare case of PNH presenting as severe dialysis-dependent AKI (serum creatinine > 2,000 μmol/L) with a positive DAT and an initially negative standard PNH screening result. This case highlights the value of renal histopathology in redirecting the diagnostic work-up and the importance of high-sensitivity FLAER-based flow cytometry when clinical suspicion persists.

## 2 Case presentation

A 60-year-old woman presented with fatigue, anorexia, loose watery stools, and dark tea-colored urine following an influenza A infection in February 2025. Initial outpatient laboratory tests indicated mild anemia, elevated creatinine (123.7 μmol/L), total bilirubin level of 76.4 μmol/L, and elevated LDH (1868 U/L). On March 11, she was admitted for suspected severe renal insufficiency. On admission, her temperature was 36.5 °C, blood pressure was 144/89 mmHg, and heart rate was 86 beats/min. Physical examination revealed pallor and mild pitting edema in both lower limbs, with no other remarkable findings. The patient’s past medical, family, and social histories were unremarkable.

Admission laboratory results on March 12 (Table 1) revealed severe AKI (creatinine 2,065 μmol/L), hemolytic anemia (hemoglobin 69 g/L, LDH 685 U/L), and a low absolute reticulocyte count (12.8 × 10^9^/L). Additional laboratory findings are summarized in Table 1. Because of severe oliguria (200 mL over 13 h), emergency hemodialysis was initiated. On March 13, the DAT was positive for anti-IgG and C3d, and the peripheral blood smear showed no schistocytes. Urinary tract ultrasonography revealed bilaterally enlarged kidneys. No thrombosis was detected in the renal artery or vein on Doppler evaluation. Further history revealed recurring episodes of dark urine since 2016, occurring intermittently and increasing in frequency over the past 2 years, with each episode typically resolving spontaneously after 2–3 days. Previous medical evaluations had failed to establish a definitive etiology.

**TABLE 1:** Clinical characteristics and laboratory findings on admission.

Urinalysis	Observed value	Reference range	Biochemistry	Observed value	Reference range
Occult blood	2+	–	Total protein	58.5	65.0–85.0 g/L
Glucose	–	–	Albumin	34.6	40.0–55.0 g/L
RBC	13.3	< 13.9/μL	LDH	685	120–250 U/L
WBC	2.3	< 13.2/μL	Total bilirubin	7.1	≤ 23.0μmol/L
uACR	235.0	≤ 30 mg/g.Cr	Uric acid	766	149–417μmol/L
uPCR	558.19	≤ 200 mg/g.Cr	BUN	65.9	3.10–8.80 mmol/L
Urobilinogen	–	–	Creatinine	2065	41–81 μmol/L
Complete blood counts			Potassium	5.81	3.5–5.3 mmol/L
WBC	5.39	3.5–9.5 × 10^9^/L	Calcium	1.88	2.17–2.75 mmol/L
RBC	2.2	3.8–5.1 × 10^12^/L	Phosphorus	1.86	0.81–1.45 mmol/L
Hemoglobin	69	115-150 g/L	Serum ferritin	1058	13–150 ng/mL
Platelets	233	125–350 × 10^9^/L	CK	33	40–200 U/L
ARC	12.8	24.0–84.0 × 10^9^/L	Immunology		
Schistocytes on blood smear	0	%	CRP	7.47	≤ 6.00 mg/L
DAT	Positive	–	ANA	–	–
PCT	0.051	< 0.052 ng/mL	anti-dsDNA	–	–
Coagulation			ANCA	–	–
PT	12.2	9.8-12.1 s	C3	1.1	0.70–1.40 g/L
APTT	29.1	23.5-36.0 s	C4	0.47	0.10–0.40 g/L
D-dimer	8.82	≤ 0.55 mg/L	IgG	8.83	8.60–17.40 g/L
ADAMTS13 activity	53.2	42.16-126.37%	IgA	1.57	1.00–4.20 g/L
			IgM	0.64	0.50–2.80 g/L

RBC, Red blood cell count; AST, Aspartate aminotransferase; WBC, White blood cell count; DAT, Direct antiglobulin test; ALT, Alanine aminotransferase; PCT, Procalcitonin; uACR, Urine albumin-to-creatinine ratio; LDH, Lactate dehydrogenase; ARC, Absolute reticulocyte count; CK, Creatine kinase; uPCR, Urine protein-to-creatinine ratio; BUN, Blood urea nitrogen; PT, Prothrombin time; CRP, C-reactive protein; APTT, Activated partial thromboplastin time; ADAMTS13, a disintegrin and metalloproteinase with thrombospondin type 1 motif 13; ANA, Antinuclear antibodies; Anti-dsDNA, Anti-double-stranded DNA; ANCA, Antineutrophil Cytoplasmic Antibodies.

**FIGURE 1:**
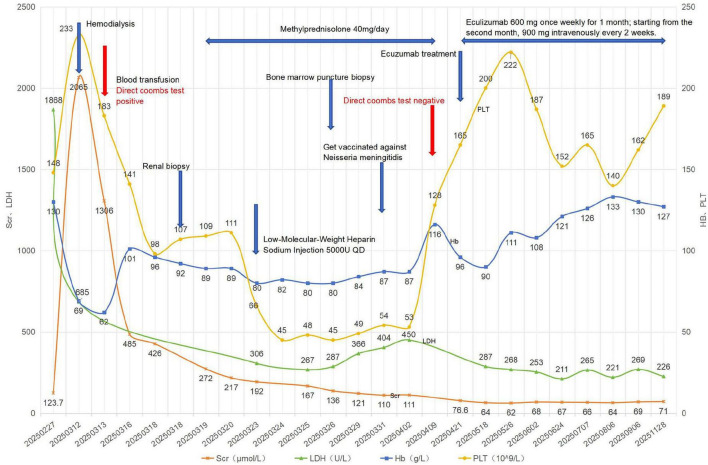
Trends in hematological and biochemical parameters during follow-up. Trends in hematological and biochemical parameters during the 7-month follow-up.

**FIGURE 2:**
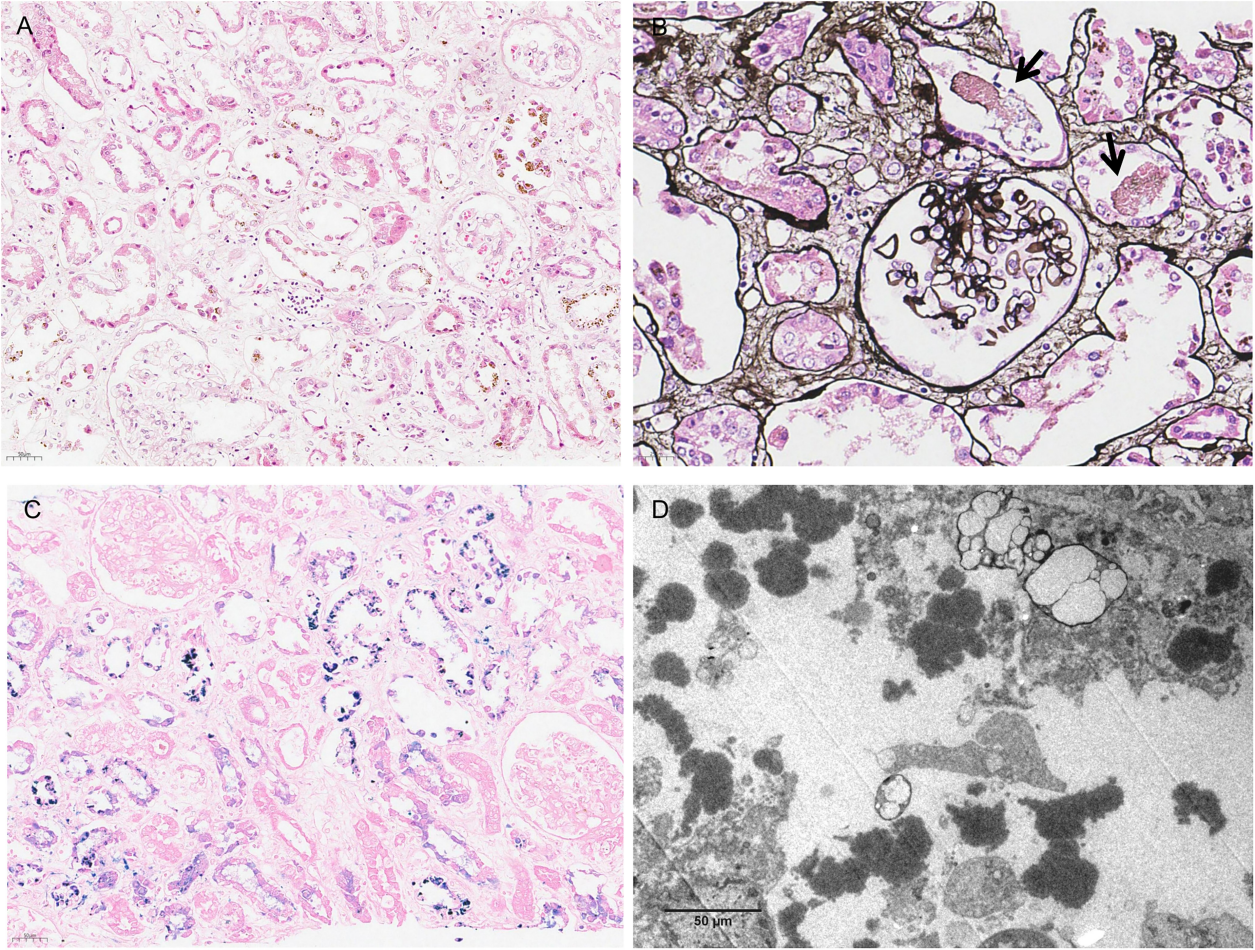
Renal biopsy findings. **(A)** Tubular epithelial necrosis and sloughing are evident, with hemosiderin granules visualized within the epithelial cytoplasm (H&E, × 200). **(B)** The glomeruli appear unremarkable. Tubular epithelial necrosis and detachment are observed, accompanied by necrotic debris within the tubular lumen (PAM, × 400). **(C)** Intracytoplasmic blue granules are demonstrated within the tubular epithelium (Prussian blue, × 200). **(D)** Electron microscopy reveals electron-dense granules located within the tubular lumen (EM, × 4,000).

The patient received 4 units of washed packed red blood cells to address anemia and underwent a course of temporary hemodialysis. Dialysis was discontinued after urine output recovered significantly, reaching 2,300 mL/day by March 16. To clarify the cause of severe AKI and ongoing hemolysis, a percutaneous renal biopsy was performed on March 18. Between March 12 and March 19, the platelet count progressively decreased (Figure 1). Normal ADAMTS13 activity and normal creatine kinase levels argued against thrombotic microangiopathy (TMA) and rhabdomyolysis, respectively. Because the DAT was positive for anti-IgG and C3d, immune-mediated hemolysis was initially considered in the differential diagnosis; however, DAT positivity alone was insufficient to establish autoimmune hemolytic anemia. In this context, intravenous methylprednisolone (40 mg/day) was empirically started on March 19 while further diagnostic evaluation was ongoing. The renal biopsy report, available on March 20, showed hemoglobin cast nephropathy with positive Prussian blue staining and thrombosis in intrarenal veins (Figure 2). Subsequent vascular ultrasound of the lower extremities revealed right iliac vein thrombosis. However, imaging also demonstrated a small perirenal hematoma secondary to the recent biopsy. Given the competing risks of thrombosis and active post-biopsy hemorrhage, prophylactic anticoagulation with low-molecular-weight heparin (5,000 IU once daily) was initiated as a temporary bridge on March 23. Following clinical confirmation of perirenal hematoma stability, therapeutic anticoagulation was successfully initiated on April 2 using oral rivaroxaban (15 mg twice daily). However, standard flow cytometry performed on March 24 showed normal CD55 and CD59 expression on red blood cells and neutrophils (Table 2).

**TABLE 2:** Results of conventional flow cytometry analysis on March 24, 2025.

Test content	Results	Reference range
Red blood cells-CD55%	99.99%	> 40.00
Red blood cells-CD59%	99.99%	> 90.00
Neutrophil granulocytes-CD55%	99.99%	> 90.00
Neutrophil granulocytes-CD59%	99.99%	> 90.00

Sample type: peripheral blood; detection method: flow cytometry; antibody: CD55, CD59.

**FIGURE 3:**
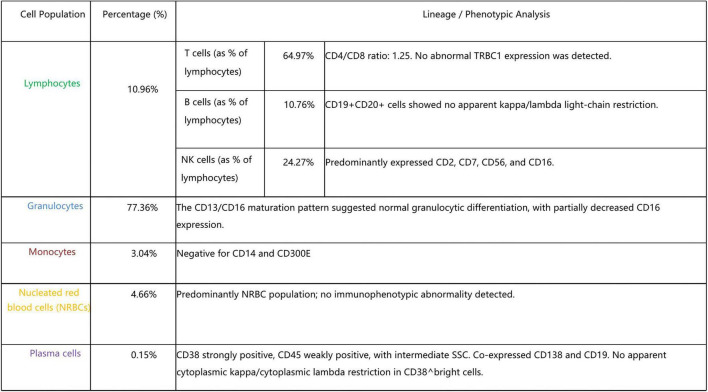
Bone marrow flow cytometry findings. Sample type: Bone marrow; Detection method: Flow cytometry. Bone marrow flow cytometry showed partial reduction of granulocyte CD16 expression and partial loss of monocyte CD14 expression, findings suggestive of PNH.

**FIGURE 4:**
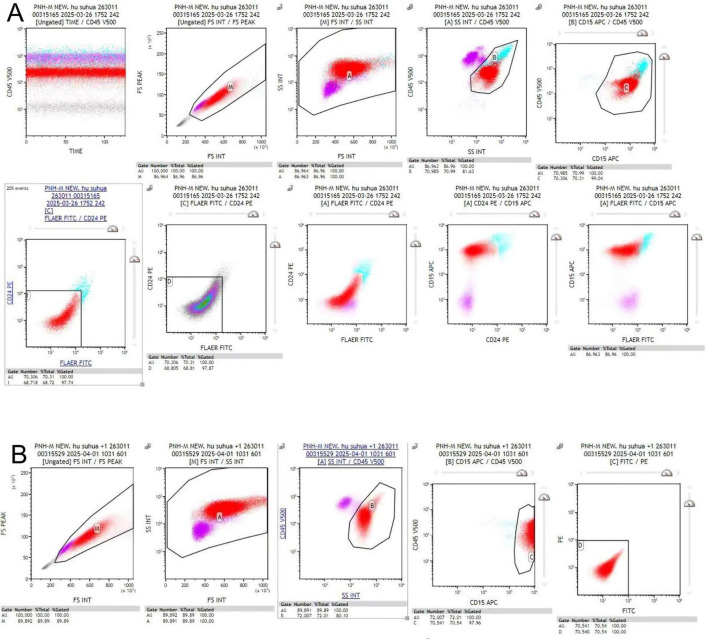
High-sensitivity FLAER-based peripheral blood flow cytometry plots. **(A)** Peripheral blood flow cytometry from the present case, demonstrating a PNH clone. **(B)** Negative control from laboratory.

In light of the observed hemolysis, low reticulocyte count, and positive DAT, further hematologic evaluation was performed. Bone marrow flow cytometry showed partial reduction of granulocyte CD16 expression and partial loss of monocyte CD14 expression (Figure 3). High-sensitivity peripheral blood flow cytometry using FLAER subsequently identified a PNH clone of 3.06% in red blood cells (type II + type III) and 98.46% in granulocytes (FLAER-CD24-) (Figure 4 and Table 3). Furthermore, targeted next-generation sequencing performed on genomic DNA extracted from unsorted peripheral blood leukocytes detected a PIGA variant, NM_002641.3 (exon2):c.548G > A (p.Cys183Tyr), with a variant allele frequency of 3.27%. Given the patient’s classic clinical phenotype and high-sensitivity flow cytometric findings, this variant was interpreted as likely pathogenic/clinically significant, firmly supporting the diagnosis of PNH. After the diagnosis was confirmed, the patient declined immediate eculizumab therapy and preferred to receive meningococcal vaccination before treatment initiation. Methylprednisolone was discontinued on April 21, and eculizumab was initiated (induction, 600 mg intravenously weekly for 4 weeks; maintenance, 900 mg every 2 weeks). During the 7-month follow-up, hemoglobin, lactate dehydrogenase, creatinine, and platelet counts gradually returned to normal (Figure 1).

**TABLE 3:** Results of high-sensitivity FLAER-based peripheral blood flow cytometry analysis on March 28, 2025.

Test content	Results
Type II red blood cells (partial deletion of CD59)	0.00%
Type III red blood cells (completely lacking CD59)	3.06%
Red blood cell PNH clone size (Type II + Type III)	3.06%
Granulocyte PNH clone size (FLAER-CD24-)	98.46%

Sample type: peripheral blood; detection method: flow cytometry; antibodies: IgG1/IgG2a, CD59/CD235a, CD45/CD15/FLAER/CD24.

## 3 Discussion

PNH is a rare acquired clonal hematopoietic disorder caused by somatic PIGA mutations, resulting in deficiency of GPI-anchored complement regulatory proteins and consequent hemolysis, thrombosis, and bone marrow failure (3, 4). Previous studies have shown that renal impairment is independently associated with an increased risk of mortality in patients with PNH, with an eightfold higher mortality rate compared with the general population (5). In addition, renal failure has been reported to account for 8–18% of deaths in patients with PNH (6).

The patient required emergency hemodialysis for severe AKI, but identifying the underlying cause remained essential. At admission, the presence of hyperbilirubinemia, elevated lactate dehydrogenase, anemia, and a positive DAT raised the possibility of immune-mediated hemolysis. The absence of schistocytes and normal ADAMTS13 activity argued against thrombotic microangiopathy. Meanwhile, the low reticulocyte count and subsequent thrombocytopenia suggested concomitant marrow involvement rather than isolated autoimmune hemolytic anemia. Notably, early standard flow cytometry showed normal CD55 and CD59 expression on red blood cells and neutrophils, posing a significant diagnostic challenge. The diagnostic breakthrough came from renal biopsy, supported by bone marrow evaluation and repeat high-sensitivity flow cytometry.

Renal injury in PNH is thought to result from multiple mechanisms, including tubular toxicity from free hemoglobin, oxidative stress, nitric oxide depletion, and thrombosis (7, 8). Histologically, hemoglobin or hemosiderin-related tubular injury is the characteristic finding, whereas glomerular changes are usually limited (9). In this case, renal biopsy demonstrated hemoglobin cast nephropathy with positive Prussian blue staining, providing important evidence of hemolysis-associated renal injury. The identification of thrombosis in intrarenal veins, together with right iliac vein thrombosis, further underscores the intrinsic thrombotic predisposition associated with PNH. The diagnosis of PNH is corroborated by positive results from high-sensitivity flow cytometry (FLAER method) and the presence of *PIGA* gene mutations (10, 11).

The discrepancy between the initial conventional CD55/CD59 assay and the subsequent FLAER-based high-sensitivity flow cytometry deserves particular attention. Several factors may account for the initially normal conventional flow cytometry result. First, the patient had received red blood cell transfusion before PNH testing, which may have diluted the proportion of GPI-deficient erythrocytes and reduced the sensitivity of red cell-based analysis (12). Second, ongoing acute hemolysis may have preferentially depleted the patient’s abnormal erythrocyte population. Third, the discordance in the granulocyte lineage reflects fundamental methodological differences. The initial conventional assay relied on secondary GPI-anchored proteins (CD55/CD59), simple CD45-based gating, and limited event acquisition. In contrast, the subsequent assay utilized FLAER—which binds directly to the GPI anchor, offering superior target specificity—combined with rigorous lineage-specific gating (e.g., CD15) and acquisition of approximately 100,000 leukocyte events. This optimized approach dramatically lowered the limit of detection, suggesting that technical and methodological differences between the assays are the most likely explanation for the initially “normal” neutrophil result. Therefore, in clinically suspected cases, a negative initial CD55/CD59 result should be interpreted with caution, and repeat testing using a high-sensitivity FLAER-based method is warranted.

Regarding the molecular findings, the PIGA variant allele frequency (3.27%) was substantially lower than the granulocyte clone size (98.46%) measured by flow cytometry. This discrepancy is primarily explained by differences in the analyzed cell populations and by the fact that variant allele frequency cannot be directly equated with clone size across different assays. Sequencing was performed on unsorted peripheral blood leukocytes, so the mutant allele was diluted by uninvolved or less-involved cell subsets, particularly long-lived lymphocytes. In contrast, high-sensitivity flow cytometry specifically quantified the GPI-deficient clone within rigorously gated granulocytes. Thus, the low variant frequency does not contradict the presence of a large granulocyte PNH clone but rather reflects assay- and specimen-related differences.

DAT positivity is uncommon but well recognized in PNH (13–16). Although PNH is classically associated with Coombs-negative hemolysis, a small proportion of patients may be DAT-positive at baseline (15). One possible explanation is complement fragment deposition on red blood cells resulting from ongoing complement activation (3, 17). In patients receiving eculizumab, inhibition of terminal complement activation does not prevent upstream C3 activation, which may lead to C3d deposition and DAT positivity (13). In the present case, recent influenza A infection may also have contributed to complement activation, although this remains speculative (18). Therefore, DAT positivity should be interpreted cautiously and should not, by itself, be taken as evidence of autoimmune hemolytic anemia when other clinical features suggest PNH.

Eculizumab remains the standard targeted therapy for PNH and was associated with hematologic remission and renal recovery in our patient (19). However, breakthrough hemolysis may still occur during long-term treatment (20).

## 4 Conclusion

This case highlights the diagnostic challenge of PNH presenting with severe AKI and a positive DAT. A positive DAT may further complicate the initial evaluation, but it neither excludes PNH nor establishes autoimmune hemolytic anemia by itself. Standard CD55/CD59 flow cytometry may yield initially negative or non-diagnostic results due to recent transfusions or methodological constraints. When clinical suspicion persists, renal histopathology, high-sensitivity FLAER-based flow cytometry, and genetic testing can be critical for establishing the diagnosis and enabling timely targeted therapy. Clinicians must maintain a high index of suspicion regarding flow cytometry confounders and utilize comprehensive testing to ensure accurate diagnosis, thereby facilitating early targeted complement inhibition to optimize both renal and hematologic outcomes.

## Data Availability

The original contributions presented in the study are included in the article/supplementary material, further inquiries can be directed to the corresponding author.
